# Durable Complete Remission of Metastatic Gastric Cancer Following Platinum-Based Chemotherapy: A Case Report

**DOI:** 10.7759/cureus.76378

**Published:** 2024-12-25

**Authors:** Weronika Pilch, Wiktoria Wojturska, Pawel M Potocki

**Affiliations:** 1 Oncology Department, Faculty of Medicine, Jagiellonian University Medical College, Cracow, POL

**Keywords:** advanced gastric cancer, capox, complete stable remission, her-2 negative, mismatch-repair proficient, pd-l1 negative

## Abstract

Gastric cancer is a common type of gastrointestinal tract malignancy. It is characterized by a poor prognosis - median survival for metastatic disease is about 12 months. A small percentage of gastric cancer is characterized by high sensitivity to systemic treatment, resulting in deep and durable responses. Predictors for such hyper-responses are still under investigation, and a wide variety of possible mechanisms exist, including the DNA damage response, intracellular signaling, immune engagement, genetic alterations, and the tumor microenvironment. Here we present a case of a 59-year-old patient with human epidermal growth factor receptor-2 (HER-2)-negative, programmed death ligand-1 (PD-L1) negative, mismatch repair proficient (pMMR) metastatic gastric cancer who reached a particularly long progression-free survival (PFS) exceeding 93 months.

## Introduction

Gastric cancer is the fifth most common neoplasm worldwide. The age-standardized incidence rate (per 100,000 population) in 2022 was 12.8 for men and 6.0 for women, whereas the global mortality rate was 8.6 and 3.9, respectively [1]. The prognosis for metastatic gastric cancer is poor. Median overall survival (OS) in a large European real-world study of patients treated in the 2010-2021 period was 3.7 months for untreated cases (~50% of cases) and 12.7 months for patients who received anti-cancer therapy [2]. There is therefore an ongoing need to develop new therapies. In phase 3 clinical trials, the combination of programmed death receptor-1 (PD-1) inhibitors with platinum doublet chemotherapy as compared to chemotherapy alone resulted in improved progression-free survival (PFS) and OS in patients with advanced gastroesophageal cancer [3-5]. Based on these studies, patients with human epidermal growth factor receptor-2 (HER-2)-negative advanced gastric cancers with high programmed death ligand-1 (PD-L1) expression should be treated with platinum-fluoropyrimidine doublet combined with PD-1 inhibitor. Whereas such patients with low PD-L1 expression should be treated with platinum-fluoropyrimidine doublet without immunotherapy. Immunotherapy-based regimen yields >20% durable responses whereas chemotherapy alone less than 5% [4]. In this case study, we present a patient with HER-2-negative, PD-L1 negative, mismatch repair proficient (pMMR) metastatic gastric cancer, who has achieved a durable complete response, following treatment with capecitabine and oxaliplatin (CAPOX) with platinum doublet chemotherapy.

## Case presentation

**Figure 1:**
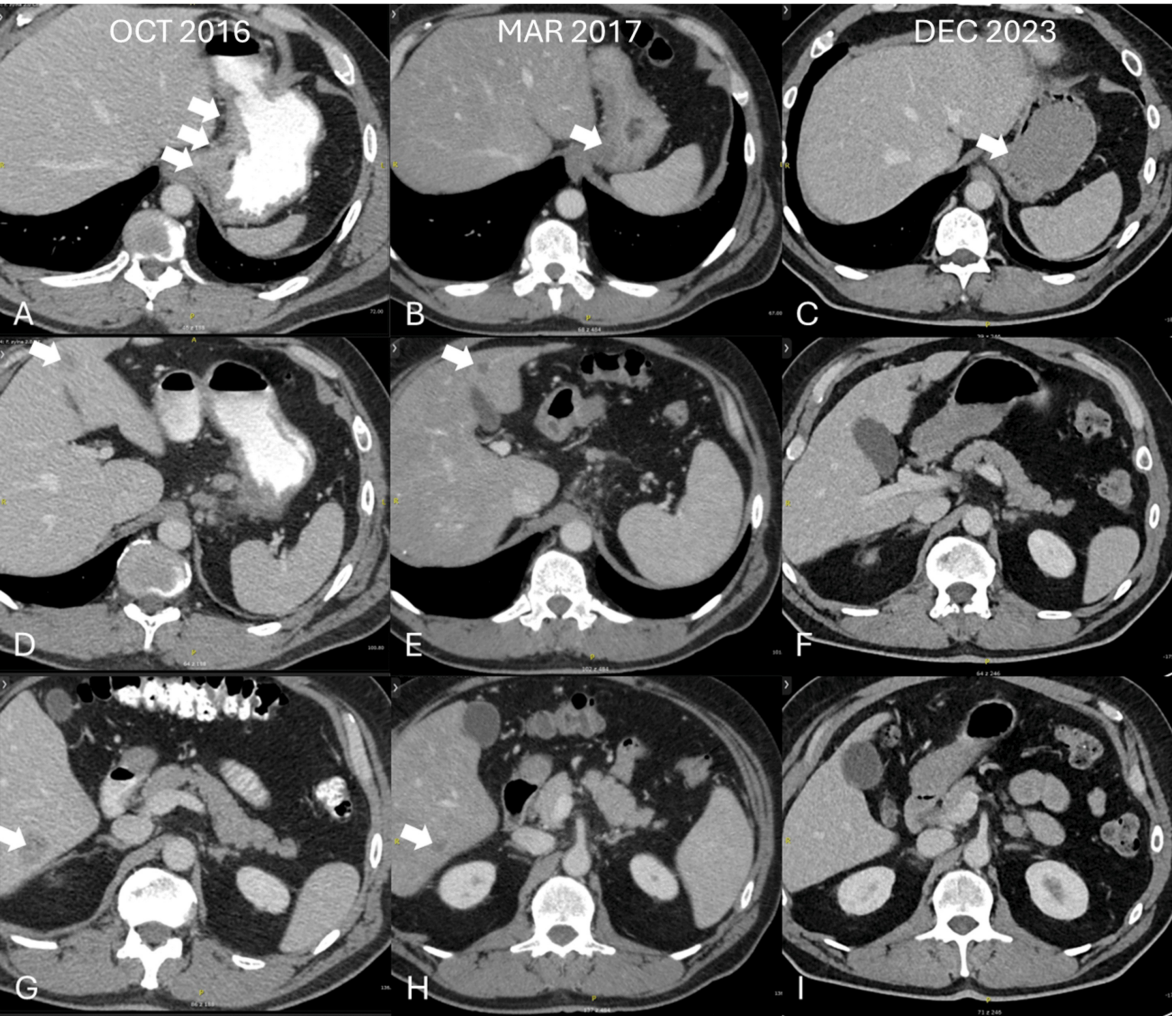
CT scans of representative cancer lesions at three timepoints. (A) The primary lesion at baseline (October 2016): contrast enhancing infiltrated cardia and enlarged regional lymph nodes; (B) the primary lesion after four months of therapy (March 2017): remission of the cardia infiltration; (C) the primary lesion at the last available follow-up scan (December 2023): no visible pathology. (D) The liver lesion in segment 4b at baseline (October 2016): peripheral contrast enhancement and hypodense center; (E) the liver lesion in segment 4b after four months of therapy (March 2017): diminished peripheral contrast enhancement and decreased diameter; (F) the liver lesion in segment 4b at the last available follow-up scan (December 2023): no visible pathology. (G) The liver lesion in segment 6 at baseline (October 2016): peripheral contrast enhancement and hypodense center; (H) the liver lesion in segment 6 after four months of therapy (March 2017): diminished peripheral contrast enhancement and decreased diameter; (I) the liver lesion in segment 6 at the last available follow-up scan (December 2023): no visible pathology. All scans presented were captured in the venous phase.

A 59-year-old patient with a month-long history of abdominal pain underwent gastroscopy in October 2016. It revealed a cancerous infiltration in the gastric antrum. Histopathological examination of the secured samples revealed a poorly differentiated tubular adenocarcinoma (no subtype). HER-2 expression was physiological (no overexpression). A post hoc additional immunohistochemical assays showed negative PD-L1 expression (a combined positive score (CPS) of less than 1), no deficiency in MMR mechanisms, and only a modest presence of tumor-infiltrating lymphocytes (~2% of the sample area). The next-generation sequencing of the tumor genome was not available. In computed tomography (CT), circular increases in antrum wall thickness (thickness: 14 mm), lymph node enlargement around the antrum, and lower curvature (short diameter: 14 mm) were shown. Furthermore, two liver lesions were identified: one in segment 4b (25 mm) and one in segment 6 (28 mm) (Figures 1A, 1D, 1G). Due to their unambiguous morphology and anatomical location, no further imaging nor biopsy verification was attempted.

In December 2016, the patient started palliative chemotherapy with the CAPOX regimen, consisting of oxaliplatin (130 mg/m^2^ on day 1) and capecitabine (2000 mg/m^2^ on days 1-14), administered every three weeks. A CT scan performed in March 2017 showed regression of lymph nodes around the antrum and a smaller curvature, as well as liver metastases (lesion in segment 4b: 9 mm, lesion in segment 6: 12 mm) (Figures 1B, 1E, 1H). Since the fifth cycle of chemotherapy, the patient had been experiencing dose-limiting myelotoxicity, resulting in a reduction in oxaliplatin dose to 75%. A subsequent CT scan (July 2017) showed a further remission in all locations. Over the course of eight months, he received nine cycles of chemotherapy, after which the decision was made to terminate treatment due to persistent hematological toxicity.

In October 2017, a follow-up CT showed stable disease with minimal increase in liver metastasis size (segment 4b lesion: 10 mm, segment 6 lesion: 14 mm). In the subsequent CT scan (January 2018), the thickness of the gastric wall was normalized and the decrease in diameter of the liver lesions (segment 4b lesion: 7 mm, segment 6 lesion: 7 mm) was observed. Since May 2018, a complete remission has been observed. A gastroscopy was performed in August 2021, which confirmed complete remission with no sign of the neoplasm, macroscopically or microscopically (in the samples secured from the initial tumor location).

The patient remains in active follow-up. The last follow-up CT scan was performed in December 2023 showing maintained complete remission (Figures 1C, 1F, 1I). As of the publication of this article, the patient has achieved a PFS of over 93 months.

## Discussion

Advanced HER-negative metastatic gastric cancer is associated with a poor prognosis [2]. Before the wide introduction of immunotherapy, the median PFS in first-line treatment with platinum-based chemotherapy of advanced gastric cancer was expected at 6.4 months, and the median OS at 11.4 months [6]. Incorporation of immune checkpoint inhibitors resulted in better, yet still unsatisfactory, results [3-5]. In the literature, case reports of exceptionally long OS have been described in metastatic gastric cancer. Ina et al. summarized cases of patients, who reached OS of at least five years and indicated high-performance status as a probable factor connected to a favorable prognosis [6]. Plausible mechanisms explaining the high chemosensitivity of various cancers include DNA damage response (DDR), intracellular signaling, immune engagement, genetic alterations, and tumor microenvironment [7]. Bilusic et al. identified DDR genes, which mutations may be related to favorable response to treatment [8]. This mechanism could be responsible for the high sensitivity to platinum-based chemotherapeutics, which are a part of the CAPOX regimen, which was administered to the presented patient [9].

Furthermore, Wheeler et al. characterized mutations that occur in exceptional responders to chemotherapy, including a patient with gastroesophageal junction carcinoma who had mutations in the TP53 p.G245S and germline EXO1 p.D249N (DDR) genes [7]. There is also an article describing a case of a particularly good response to treatment in a patient with metastatic gastric cancer with the PALB2 gene mutation [10]. This mutation is associated with homologous recombination deficiency and can lead to high sensitivity to platinum-based agents [10]. Unfortunately, the authors were unable to perform molecular profiling of cancer tissue in search of those biomarkers in the case described here, therefore, the underlying reason behind the achieved results remains uncertain.

In the literature from recent years, immunotherapy has been shown to be a promising treatment method for advanced gastric cancer [10]. Phase 3 clinical trial KEYNOTE-859 determined that patients receiving chemotherapy with a PD-1 inhibitor, pembrolizumab, had longer OS and PFS in comparison to patients receiving chemotherapy alone (OS: 12.9 months vs 11.5 months; p<0.0001; PFS: 6.9 months vs 5.6 months; p<0.0001) [3]. Furthermore, in the CheckMate 649 trial, OS and PFS were significantly longer in a group treated with chemotherapy and nivolumab than in a group treated with only chemotherapy (OS: hazard ratio (HR): 0.71; 98.4% confidence interval (CI): 0.59-0.86; p<0.0001; PFS: HR: 0.68; 98% CI: 0.56-0.81; p<0.0001) [4]. In both studies, the presence of high PD-L1 expression and the presence of MMR deficiency were, respectively, moderate and a strong predictive factor for response. Checkpoint inhibitor immunotherapy is now incorporated into clinical practice guidelines for patients with high expression of PD-L1 [11].

## Conclusions

The patient presented in this case achieved what can be considered a cure for metastatic gastric cancer. The underlying mechanisms that allowed for such a remarkable response remain unknown. Known standard prognostic and predictive factors including histological subtype, HER-2, PD-L1, and MMR were negative. Recent advances in systemic therapy of advanced gastric cancer increased the odds of achieving a durable response, yet the predictive factors for such a result remain elusive. More studies are needed to identify the mechanisms that will allow achieving a greater antitumor response and better survival of patients.
